# Stress Alters the Effect of Alcohol on Catecholamine Dynamics in the Basolateral Amygdala

**DOI:** 10.3389/fnbeh.2021.640651

**Published:** 2021-04-15

**Authors:** Alex L. Deal, Jinwoo Park, Jeff L. Weiner, Evgeny A. Budygin

**Affiliations:** ^1^Department of Neurobiology and Anatomy, Wake Forest School of Medicine, Winston-Salem, NC, United States; ^2^Department of Biotechnical and Clinical Laboratory Sciences, University at Buffalo, State University of New York, Buffalo, NY, United States; ^3^Department of Pharmacology and Toxicology, University at Buffalo, State University of New York, Buffalo, NY, United States; ^4^Department of Physiology and Pharmacology, Wake Forest School of Medicine, Winston-Salem, NC, United States

**Keywords:** norepinephrine, dopamine, FSCV, fast-scan cyclic voltammetry, locus coeruleus (LC), social defeat stress, forced swim test (FST)

## Abstract

The current rodent study applied *in vivo* fast-scan cyclic voltammetry (FSCV), paired with a pharmacological approach, to measure the release of the catecholamines (CA) dopamine (DA) and norepinephrine (NE) in the basolateral amygdala (BLA) following locus coeruleus (LC) stimulation. The primary goal was to determine if exposure to either social (social defeat) or non-social (forced swim) stress altered LC-evoked catecholamine release dynamics in the BLA. We used idazoxan (α2 adrenergic receptor antagonist) and raclopride (D_2_ dopamine receptor antagonist) to confirm the presence of NE and DA, respectively, in the measured CA signal. In non-stressed rats, injection of idazoxan, but not raclopride, resulted in a significant increase in the detected CA signal, indicating the presence of NE but not DA. Following exposure to either stress paradigm, the measured CA release was significantly greater after injection of either drug, suggesting the presence of both NE and DA in the LC-induced CA signal after social or non-social stress. Furthermore, acute administration of alcohol significantly decreased the CA signal in stressed rats, while it did not have an effect in naïve animals. Together, these data reveal that, while LC stimulation primarily elicits NE release in the BLA of control animals, both social and non-social stress unmask a novel dopaminergic component of LC catecholamine signaling. Future studies will be needed to identify the specific neural mechanism(s) responsible for these plastic changes in LC-BLA catecholamine signaling and to assess the possible contribution of these changes to the maladaptive behavioral phenotypes that develop following exposure to these stressors.

## Introduction

Mood and anxiety disorders are highly comorbid with alcohol use disorder (AUD; Grant et al., 2004). For example, those diagnosed with post-traumatic stress disorder are three times more likely to develop an AUD (Kessler et al., 1997). Even in individuals without a formal diagnosis, alcohol is a common tool for those with self-reported social anxiety to cope with stressful situations. These people are more likely to consume alcohol to feel more comfortable socially (Thomas et al., 2003). Despite the strong relationship between stress and alcohol, much remains unknown about how one can alter the neurobiological consequences of the other.

The locus coeruleus (LC) is a key brainstem nucleus involved in stress and arousal (Szabadi, 2013; Naegeli et al., 2018; Daviu et al., 2019). This pontine structure is the primary source of noradrenergic projections in the central nervous system which target many regions throughout the brain, such as the amygdala, thalamus, hypothalamus, and prefrontal cortex, associating the LC with myriad behavioral, motor, and sensory processes (Samuels and Szabadi, 2008; Deal et al., 2019). In particular, the projection from the LC to the basolateral amygdala (BLA) has been proposed as an important pathway in anxiety-related processes and behaviors. Recent work has shown that optogenetic activation of this pathway produced anxiety-like behavior in rats as demonstrated by a reduction in time spent in the center of an open field test and a reduction in time spent in the open portions of an elevated zero maze (McCall et al., 2017). Moreover, it was recently revealed that the LC-norepinephrine (NE) circuitry can bidirectionally control alcohol-drinking behaviors through different patterns of optoactivation (Deal A. L. et al., 2020).

On other hand, stress has been shown to be a key influencer of catecholamine (CA) activity and dynamics throughout the brain (Tanaka et al., 1991; Ferry et al., 1999; Inglis and Moghaddam, 1999; Anstrom et al., 2009; Karkhanis et al., 2015; Rajbhandari et al., 2015; Holly and Miczek, 2016; Deal et al., 2018; Giustino et al., 2020). For example, studies using *ex vivo* fast-scan cyclic voltammetry (FSCV) have reported that adolescent social isolation stress (Yorgason et al., 2013, 2016) as well as social defeat stress (Deal et al., 2018) resulted in increased dopamine (DA) release and its consequent reuptake in striatal subregions. Furthermore, social isolation stress during adolescence was found to elicit lasting changes to DA and DA transporters in the BLA, specifically a decrease in DA levels as measured by microdialysis and an increase in DA transporters in adulthood (Karkhanis et al., 2015). These findings are further reinforced by *in vivo* voltammetric experiments demonstrating an increased uptake rate and decreased release of NE in the bed nucleus of the stria terminals in rats who experienced social isolation (Fox et al., 2015). Notably, no studies have examined post-stress catecholamine dynamics within the LC-BLA circuitry or whether stress-related alterations in this pathway alter the effects of alcohol on DA and NE transmission.

To explore these questions, we used *in vivo* FSCV, a methodology that permits the real-time detection of extracellular DA and NE concentrations at terminals following electrical stimulation of cell body regions (Budygin et al., 2000, 2007, 2017; Mateo et al., 2004; Oleson et al., 2009; Park et al., 2011, 2012; Fox et al., 2015, 2016). This approach allowed us to examine the effects of two different stressors, repeated social defeat stress (RSDS) and forced swim test (FST), on LC-evoked CA release in the BLA and the sensitivity of the CA signal to acute alcohol. These stress paradigms have been shown to significantly elevate serum corticosterone levels in rats, a key marker of stress circuitry activity (Báez and Volosin, 1994; Miczek et al., 1999; Covington and Miczek, 2005; Barnum et al., 2007; Hueston et al., 2011; Moravcova et al., 2021). After first confirming the CA signal, we pharmacologically identified the components of the CA signal in stressed and non-stressed subjects as NE or DA by injecting either idazoxan (α_2_ adrenergic receptor antagonist) or raclopride [dopamine (DA) D_2_ receptor antagonist], respectively, and determined whether the evoked CA signal was altered by acute alcohol.

## Materials and Methods

### Animals

Adult male Sprague–Dawley rats (300–350 g) were used as intruder (stressed) subjects and retired breeder male Long Evans rats (>450 g) served as resident (aggressor) rats (Wood et al., 2010, 2013; Arora et al., 2019). The utilization of different strains resulted in a more robust stress environment due to the aggressive nature of the Long Evans rat (Henry et al., 1993; Snyder et al., 2018). A total of 28 Sprague–Dawley and 6 Long Evans rats were used during this study. Animals were kept on a 12-h light/dark cycle with food and water available *ad libitum*. Animal handling and all procedures were conducted in accordance with the National Institutes of Health Guide for the Care and Use of Laboratory Animals. All procedures were approved by the Wake Forest University School of Medicine Institutional Animal Care and Use Committee (protocol A19-190).

### Repeated Social Defeat Stress

**Figure 1:**
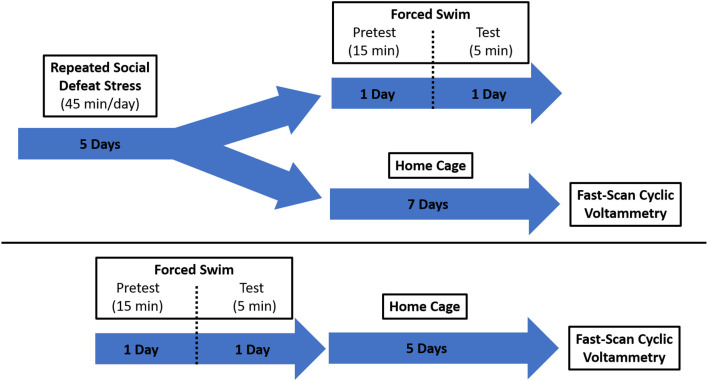
Schematic showing the experimental design. Two groups of rats were exposed to different stress paradigms, social defeat and forced swim. They were then evaluated for protracted effects of stress on catecholamine dynamics using fast-scan cyclic voltammetry (FSCV).

Intruder rats were exposed to five 45-min social defeat sessions prior to use for voltammetric recordings (or remained in their home cage to serve as a control). For five consecutive days, intruders were acclimated for 1 h to the testing room and then placed in the cage of a larger, aggressive resident male (see Figure 1 for experimental design). The rats were allowed to physically interact for 15 min. If blood was observed following a physical bout, the injury was assessed by an observing experimenter for severity. Minor injury resulted in immediate transferal to a protective mesh cage within the resident cage for the remainder of the session. Major injuries, those requiring suturing or other invasive interventions, would have resulted in removal of the intruder rat from the study (no major injuries occurred and thus no rats were excluded from the experiment). Typical sessions included several aggressive physical interactions that resulted in the intruder rat exhibiting subordinate behavior through assuming a supine position. At the conclusion of the physical interaction time, the remainder of the session (30 min) took place while the intruder was protected within a wire mesh cage inside of the resident cage. Visual, auditory, and olfactory signal exposure was still possible during this time.

### Forced Swim Test

Rats were forced to swim as described previously (Porsolt et al., 1978). Briefly, animals were allowed to acclimate to the testing room for 1 h and then placed in a transparent acrylic cylinder (30 × 50 cm) filled with 40 cm of water (25 ± 1°C) for 15 min (Linthorst et al., 2008; Bogdanova et al., 2013). One day later, the rats were placed in the water-filled cylinder again for 5 min. The rats were allowed to dry off in their home cage, which was placed on a heating pad, for at least 1 h before the cage was returned to the colony room. The cylinder was cleaned with soap and water after each rat.

A separate group of RSDS rats underwent the FST procedure immediately following the last day of social defeat exposure to behaviorally verify a stress-like phenotype. These rats were not included in subsequent voltammetry experiments. Video recordings were made for each rat on both days of FST exposure. Day 2 (test day) recordings were analyzed by an experimenter blinded to the condition and scored for bouts of immobility. The experimenter evaluated behavior every 5 s for the duration of the test (5 min) as either swimming or immobile (Deal A. W. et al., 2020).

### Fast-Scan Cyclic Voltammetry

Rats were taken 1 week after the final defeat session or 5 days after the second forced swim session, anesthetized *via* urethane injection (1.35 g/kg, i.p.), and secured in a stereotaxic frame. Holes were drilled to allow for the placement of a carbon fiber recording electrode in the basolateral amygdala (from bregma: AP −2.9; ML + 4.6; DV −8.0), a bipolar stimulating electrode in the ipsilateral locus coeruleus (AP −9.8; ML + 1.3; DV −7.2), and a Ag/AgCl reference electrode in the contralateral hemisphere. The reference electrode was connected to a voltammetric amplifier (UNC Electronics Design Facility, Chapel Hill, NC, USA). The carbon fiber microelectrode (exposed fiber length: 75–100 μm; diameter: 6 μm) was connected to the voltammetric amplifier and secured to the stereotaxic frame arm. Stimulations were made every 10 min by an electrical pulse. Extracellular CA was recorded at the carbon fiber electrode every 100 ms for 15 s by applying a triangular waveform (−0.4 V to +1.3 V and back to −0.4 V vs Ag/AgCl, 400 V/s). The catecholamine signal was identified by observing background-subtracted cyclic voltammograms characterized by oxidation and reduction peaks occurring at +0.6 and −0.2 V, respectively. A stable baseline signal was established (three consecutive recordings within 10% variability) and an average baseline value was calculated. Immediately after establishing a baseline, an injection (i.p.) of saline, raclopride (dopamine D_2_ receptor antagonist; 2 mg/kg), idazoxan (α_2_ adrenergic receptor antagonist; 5 mg/kg), or alcohol (2 g/kg ethanol) was administered. These idazoxan and raclopride doses effectively increase an electrically-evoked catecholamine efflux clarifying whether DA (raclopride) or NE (idazoxan) is detected in the rat brain with FSCV (Park et al., 2015; Fox et al., 2016; Mikhailova et al., 2019; Deal et al., 2018; Deal A. L. et al., 2020). Some rats received more than one drug during the FSCV experiment. In such cases, the CA signal was allowed to completely return to pre-injection levels before a new baseline was established. Additionally, the order of injections was balanced to prevent any confounding effects of drug interactions. Voltammetric recordings were taken for 60 min after injection. Data were digitized (National Instruments, Austin, TX, USA) and stored on a computer. Each carbon fiber electrode was calibrated after use in an experiment in a flow injection analysis system. Calibrations were performed in triplicate using a known concentration (10 μM) of DA (Sigma–Aldrich, St. Louis, MO, USA) and calibrated again using a known concentration (10 μM) of NE (Sigma–Aldrich, St. Louis, MO, USA). The voltammetric current was measured at the peak potential.

### Histology

**Figure 2:**
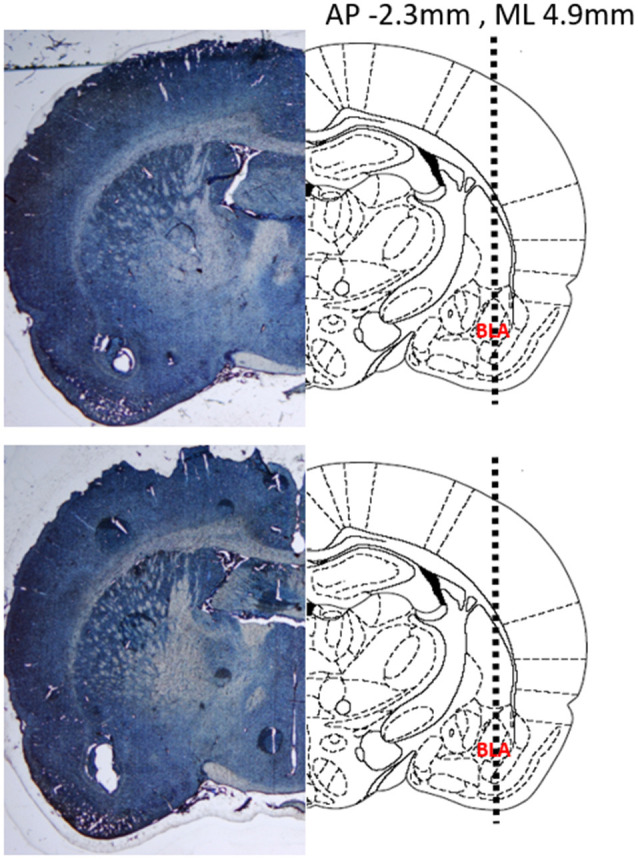
Representative coronal sections demonstrating the location of the recording electrode in two different rat brains. (Left) Thionine stained brain slices showing a lesion at the recording site in the basolateral amygdala (BLA). (Right) Atlas diagrams with the BLA labeled and a dashed line showing the insertion trajectory of the recording electrode. Coordinates provided are relative to Bregma. Diagram adapted from Paxinos and Watson (2004).

Verification of electrode placements was performed as previously described (Park et al., 2010; Budygin et al., 2012). Briefly, a constant current (20 μA, 10 s) was applied to the carbon fiber electrode to create a lesion at the site of recording. Brains were removed and submerged in 10% formaldehyde for a minimum of 3 days. After adequate fixation, the brains were coronally sliced at a thickness of 40–50 μm using a cryostat. Once the slices were mounted on slides, they were stained with 0.2% thionine and cover slipped before imaging was performed using a light microscope. Representative slices from two different rats demonstrating lesions at the site of the recording electrode are shown with accompanying atlas diagrams (Figure 2).

### Data Analysis

Data were analyzed using GraphPad Prism (GraphPad Software version 7.04, San Diego, CA, USA). Unpaired *t*-test, repeated measures two-way ANOVAs, and Tukey’s multiple comparisons tests were used to analyze data where appropriate. Data are presented as mean ± SEM and the criterion for significance was set at *p* < 0.05.

## Results

### Electrical Stimulation of LC Elicits NE Release in the BLA

**Figure 3:**
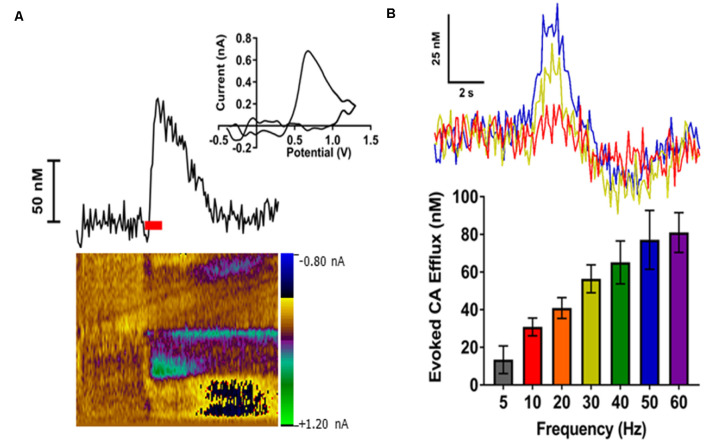
Electrical stimulation of the locus coeruleus (LC) reliably evoked catecholamines (CA) release in the BLA in a frequency-dependent manner. **(A)** A representative trace showing changes in CA concentration over time (top left). The respective cyclic voltammogram (top right) and color plot (bottom) are also shown. The red bar indicates duration of electrical stimulus. **(B)** Representative concentration-time traces of CA release (top) in the BLA of an individual rat demonstrate an increased response as stimulation frequency increases (Red = 10 Hz; Yellow = 30 Hz; Blue = 50 Hz). Averaged CA responses (bottom) show higher frequency stimulations resulted in increased efflux concentrations compared to lower frequency stimulations. Data are presented as mean ± SEM.

**Figure 4:**
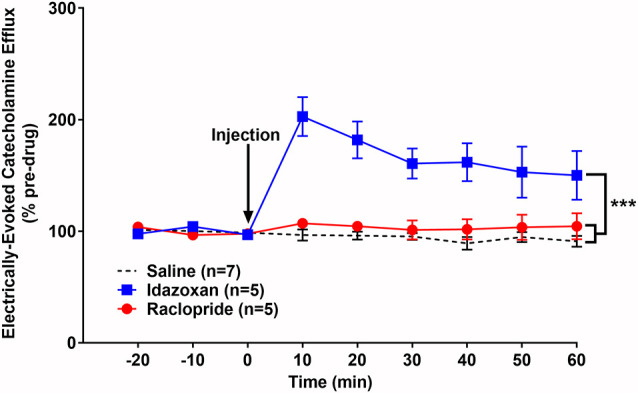
Idazoxan significantly increased norepinephrine (NE) in the basolateral amygdala (BLA) following locus coeruleus (LC) electrical stimulation in non-stressed rats. Anesthetized rats were used to quantify LC-evoked CA release in the BLA, as measured by fast-scan cyclic voltammetry. After a stable baseline was established, saline, idazoxan, or raclopride was administered (black arrow). Idazoxan resulted in a significant increase in the measured signal compared to the saline control. There was no change in the CA signal following injection of raclopride compared to the saline control. Efflux values are presented as mean (± SEM) percent of a pre-injection baseline average. Saline: *n* = 7; Idazoxan: *n* = 5; Raclopride: *n* = 5. ***p < 0.001.

We have previously shown that optogenetic activation of the LC results in a robust noradrenergic signal in the BLA, as detected by FSCV (Deal A. L. et al., 2020). Here, we evoked release by electrical LC stimulation that resulted in a consistent, robust, frequency-dependent signal in the BLA which matched the characteristics of CA release (Figure 3). Next, in order to identify the constitutive components of this signal, a pharmacological approach was used. More specifically, doses of idazoxan (*n* = 5) or raclopride (*n* = 5) were administered to determine the presence of norepinephrine or dopamine, respectively, compared to a saline control (*n* = 7; Figure 4). A repeated measure two-way ANOVA found the main effect of drug (*F*_(2, 14)_ = 20.28; *p* < 0.0001), time (*F*_(8, 112)_ = 9.354; *p* < 0.0001), and a significant interaction (*F*_(16, 112)_ = 9.101; *p* < 0.0001). A follow-up *post hoc* Tukey’s multiple comparison test found that idazoxan significantly increased the measured CA signal compared to saline (*p* < 0.0001) and raclopride (*p* < 0.001) and there was no significant difference after raclopride injection (*p* = 0.718). These results demonstrate that NE is the predominant CA evoked in the BLA following LC electrical stimulation.

### Electrical LC Stimulation in Socially Stressed Subjects Elicits NE and DA Release in BLA

To determine the effect of stress on evoked CA transients in the BLA, intruder rats were used for *in vivo* FSCV for 1 week following the last 5 days of repeated social defeat stress. In a separate group of rats, we verified that the social defeat procedure resulted in stress by exposing RSDS rats to a forced swim test. A one-tailed unpaired *t*-test found a significantly higher average number of bouts of immobility exhibited by rats exposed to RSDS (11.1 ± 1.65; *n* = 5) compared to non-stressed controls (7.21 ± 1.32; *n* = 7; *t*_(10)_ = 1.854; *p* < 0.05) suggesting a stress-like phenotype.

**Figure 5:**
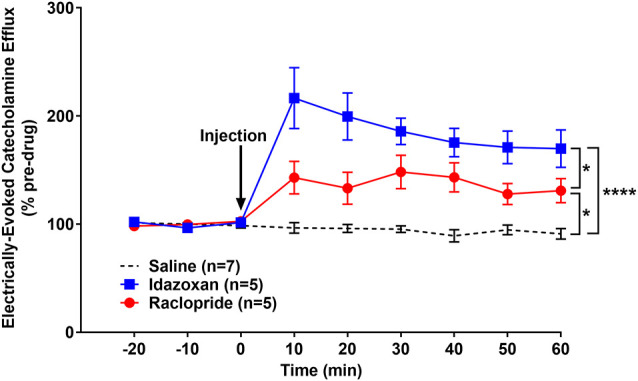
Idazoxan and raclopride significantly increased norepinephrine and dopamine (DA), respectively, in the basolateral amygdala (BLA) following locus coeruleus (LC) electrical stimulation in rats exposed to repeated social defeat stress (RSDS). Rats exposed to 5 days of social defeat stress were anesthetized and used to quantify LC-evoked CA release in the BLA, as measured by fast-scan cyclic voltammetry. After a stable baseline was established, saline, idazoxan, or raclopride was administered (black arrow). Idazoxan and raclopride resulted in a significant increase in the measured signal compared to the saline control. Efflux values are presented as mean (± SEM) percent of a pre-injection baseline average. Saline: *n* = 7; Idazoxan: *n* = 5; Raclopride: *n* = 5. *p < 0.05; ****p < 0.0001.

Similar to the naïve group, idazoxan (*n* = 5) and raclopride (*n* = 5) were administered to determine the catecholamines (CA) present in the detected signal in rats exposed to RSDS and compared to a saline control (*n* = 7; Figure 5). A repeated measure two-way ANOVA found the main effect of drug (*F*_(2, 14)_ = 22.77; *p* < 0.0001), time (*F*_(8, 112)_ = 20.02; *p* < 0.0001), and a significant interaction (*F*_(16, 112)_ = 9.855; *p* < 0.0001). A *post hoc* Tukey’s multiple comparisons test showed that injection of either idazoxan or raclopride significantly increased the evoked CA signal compared to saline (*p* < 0.0001 and *p* = 0.0164, respectively) and idazoxan increased the signal significantly more than raclopride (*p* = 0.0145). These data show that electrical stimulation of the LC evokes both NE and DA release in the BLA of subjects exposed to repeated social defeat stress.

### Exposure to Forced Swim Stress Results in NE and DA Release in the BLA Following LC Stimulation

**Figure 6:**
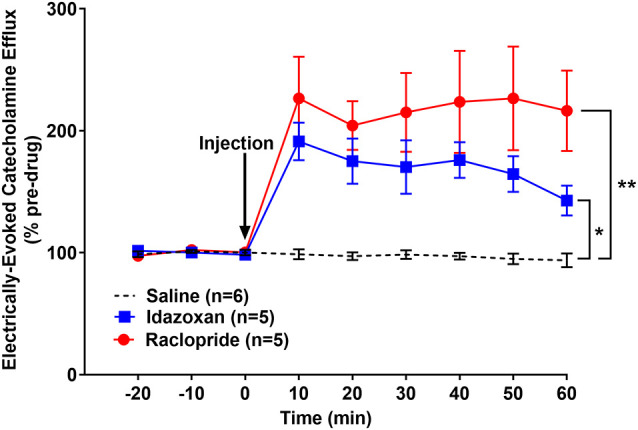
Idazoxan and raclopride significantly increased norepinephrine and dopamine, respectively, in the basolateral amygdala (BLA) following locus coeruleus (LC) electrical stimulation in rats exposed to a forced swim test (FST). Rats exposed to a forced swim test were anesthetized and used to quantify LC-evoked CA release in the BLA, as measured by fast-scan cyclic voltammetry. After a stable baseline was established, saline, idazoxan, or raclopride was administered (black arrow). Idazoxan and raclopride resulted in a significant increase in the measured signal compared to the saline control. Efflux values are presented as mean (± SEM) percent of a pre-injection baseline average. Saline: *n* = 6; Idazoxan: *n* = 5; Raclopride: *n* = 5. *p < 0.05; **p < 0.01.

In order to determine if this effect of social stress on CA release was unique or generalizable to other stressors, a separate cohort of naïve rats (not exposed to RSDS) was exposed to FST and then used for *in vivo* FSCV (Figure 6). A repeated measures two-way ANOVA calculated the main effect of drug (*F*_(2, 13)_ = 10.3; *p* < 0.01), time (*F*_(8, 104)_ = 21.26; *p* < 0.0001), and a significant interaction (*F*_(16, 104)_ = 7.489; *p* < 0.0001). A *post hoc* Tukey’s multiple comparisons test found that both idazoxan (*n* = 5) and raclopride (*n* = 5) increased the measured CA release compared to saline controls (*n* = 6; *p* = 0.0458 and *p* < 0.01, respectively). Taken together these experiments suggest that stress, either social and non-social, results in the release of DA, in addition to NE, in the BLA following LC electrical stimulation.

Interestingly, when compared across stress paradigms, raclopride administration resulted in a significantly higher efflux of dopamine following FST compared to either RSDS or non-stressed, naïve controls (*n* = 5 for all groups; two-way RM ANOVA: drug, *F*_(3, 18)_ = 12.26, *p* < 0.0001; time, *F*_(8, 144)_ = 14.36, *p* < 0.0001; interaction, *F*_(24, 144)_ = 7.814, *p* < 0.0001; Tukey’s multiple comparisons test: *p* < 0.05). In a similar comparison, injection of idazoxan did not result in a significant difference between the stress and control groups (*n* = 5 for all groups; two-way RM ANOVA: drug, *F*_(3, 18)_ = 13.32, *p* < 0.0001; time, *F*_(8, 144)_ = 41.71, *p* < 0.0001; interaction, *F*_(24, 144)_ = 6.848, *p* < 0.0001; Tukey’s multiple comparisons test: *p* > 0.05) This suggests that there are possible stressor-specific effects on dopamine release which should be considered when using these different paradigms.

### Stress Exposure Does Not Alter LC-Evoked NE in the BLA

Additional analysis of the signal prior to injection of saline or drug was conducted to determine if stress exposure altered NE release. A one-way ANOVA found that there was no difference in NE release between the non-stressed (*n* = 5; 251.2 ± 62.6), RSDS (*n* = 8; 214.7 ± 30.2), and FST subjects (*n* = 6; 184.9 ± 29.6; *F*_(2, 16)_ = 0.617; *p* = 0.552). However, though not significant, some tendency towards reduced electrically-evoked NE release following forced swim test exposure was evident. A comparison of DA release was not possible because raclopride did not significantly alter the CA signal in non-stressed subjects thus suggesting DA was not present (Figure 4).

### Alcohol Decreases the Evoked CA Signal in the BLA by LC Stimulation in Stressed Subjects

**Figure 7:**
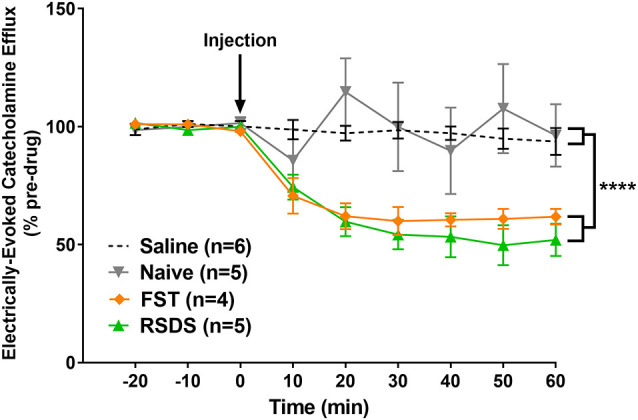
Alcohol significantly reduced the measured catecholamine (CA) efflux in the basolateral amygdala (BLA) following locus coeruleus (LC) electrical stimulation of stressed subjects. Rats were exposed to either repeated social defeat stress or the forced swim test prior to being used to quantify LC-evoked CA release in the BLA as measured by fast-scan cyclic voltammetry. After a stable baseline was established, alcohol was injected (black arrow). In stressed subjects, those exposed to either repeated social defeat stress (RSDS) or forced swim test (FST), alcohol resulted in a significant decrease in the measured CA signal compared to non-stressed, naïve controls. Efflux values are presented as mean (± SEM) percent of a pre-injection baseline average. Saline: *n* = 6; Naïve: *n* = 5; FST: *n* = 4; RSDS: *n* = 5. ****p < 0.0001.

In order to explore whether stress is capable of altering the effect of alcohol on CA dynamics, ethanol (2 g/kg, i.p.) was acutely administered to anesthetized subjects previously exposed to RSDS (*n* = 5) or FST (*n* = 4; Figure 7). A repeated measures, two-way ANOVA was conducted and found a main effect of condition (*F*_(3, 16)_ = 8.408; *p* < 0.001), time (*F*_(8, 128)_ = 11.79; *p* < 0.0001), and a significant interaction (*F*_(24, 128)_ = 4.026; *p* < 0.0001). A *post hoc* Tukey’s multiple comparisons test found that alcohol attenuated CA release in both RSDS and FST groups compared to saline (*p* < 0.01 and *p* = 0.0309, respectively) and non-stressed controls (*p* < 0.01 and *p* = 0.0262, respectively). In addition, there was no significant change following alcohol injection in control animals compared to saline (*p* > 0.05). These data show that alcohol decreases LC-evoked BLA CA release in subjects exposed to stress but does not have an effect in non-stressed controls.

## Discussion

These experiments demonstrate robust stress-dependent alterations in LC-evoked CA release in the BLA following LC stimulation. In control subjects, LC electrical stimulation elicits a strong, predominantly noradrenergic signal in the BLA whose efflux is not altered by acute alcohol. However, in animals that have been exposed to stress, through either social defeat or forced swim, electrical stimulation of the LC results in a CA efflux that is mediated by both noradrenergic as well as dopaminergic components and this response is attenuated following administration of alcohol. Taken together, these findings suggest a role for stress in altering catecholamine dynamics in the BLA and a change in the sensitivity of evoked CA to alcohol.

Although NE release evoked by LC stimulation was previously detected with *in vivo* FSCV in the prefrontal cortex and bed nucleus of the stria terminals (Park et al., 2011, 2012, 2013; Fox et al., 2016; Deal et al., 2019), our results for the first time demonstrate the possibility to measure real-time NE efflux in the BLA. Based on pharmacological characterizations, we ruled out the involvement of DA efflux in the signal measured in naïve rats as previously reported for the prefrontal cortex and bed nucleus of the stria terminals (Park et al., 2011; Fox et al., 2016; Deal et al., 2019). As expected, the electrically-evoked NE release was frequency-dependent, while the average maximum concentrations were ~2–3 fold lower than concentrations in the prefrontal cortex (Deal et al., 2019). An intriguing finding was the raclopride-induced increase in the detected CA signal in subjects following exposure to both stress paradigms. These data clearly indicate the appearance of a DA component that was not detected in control animals. The origins of the evoked DA signal are unclear and therefore should be further explored. However, some speculations can be offered.

A major dopaminergic source in the brain is the ventral tegmental area (VTA), which has connections with the LC and BLA (Shelkar et al., 2017; Breton et al., 2019). Indeed, social defeat stress has been shown to increase phasic DA release in the nucleus accumbens (NA), a region with strong dopaminergic input from the VTA (Anstrom et al., 2009). Further, chronic stress exposure can lead to morphological changes in VTA DA neurons and enhanced excitability of this neuronal population (Douma and de Kloet, 2020). Perhaps a stress-promoted increase in the excitability of VTA dopaminergic neurons could result in the triggering of DA release in the BLA in response to LC stimulation. Therefore, one hypothesis to address the current findings is that the stress-triggered DA portion of the evoked CA signal reflects a persistent increase in the excitability of VTA projections.

Stress has also been shown to alter the electrophysiological characteristics of LC neurons (Jedema and Grace, 2003; Borodovitsyna et al., 2018). Previous studies have reported that exposure of adolescent rats to restraint and predator odor stressors resulted in increased excitability and spontaneous discharge of LC neurons, which were evident 1 week after stress exposure (Borodovitsyna et al., 2018). Thus, it is possible that in the present study, forced swim or repeated social defeat stress induced a similar increase in LC neuron excitability, altering the activation dynamics of LC inputs to VTA DA neurons, resulting in amygdalar DA release. Therefore, another possible origin for the DA component in the CA signal of stressed subjects is from changes to the NE neurons of the LC that innervate the VTA. In fact, a third possibility can combine both mentioned above scenarios.

Finally, multiple studies have found that various stressors result in an increased expression of tyrosine hydroxylase (TH) mRNA in LC neurons (Mamalaki et al., 1992; Rusnák et al., 2001). While these studies did not confirm the lasting effects of stress on TH mRNA, such an increase in this rate-limiting enzyme of DA synthesis could result in elevated levels of DA in LC NE neurons. Supporting this possibility, another study found that chronic social defeat stress resulted in an upregulation of dopamine β-hydroxylase (DBH) mRNA and protein in the LC as well as increased DBH protein levels in terminal regions of the LC, such as the amygdala, hippocampus, and frontal cortex (Fan et al., 2013). The increase in the production of this ezyme that converts DA to NE can be induced by accumulation of DA inside of NE neurons. Consequently, LC stimulation of stressed rats could result in the efflux of both DA and NE from noradrenergic projections to the BLA due to elevated levels of DA production within the LC NE neurons. Though not induced by stress, there is evidence to support the release of DA from noradrenaline neurons in some brain areas, including prefrontal cortex and hippocampus (Devoto et al., 2003, 2019; Kempadoo et al., 2016).

The finding that consequences of social and non-social stress on CA dynamics are not different is somewhat surprising. However, it is important to highlight that our experiments focused on evoked NE and DA releases which are not necessarily correlated with basal activity of neurotransmitters. Therefore, this question requires further exploration with complimentary techniques which can evalutate changes in other modes of CA neurotransmission.

Another stress-induced change in the CA signal was observed following the administration of alcohol. In non-stressed animals, alcohol did not significantly alter the measured CA efflux. However, following stress exposure, alcohol attenuated the evoked CA signal in the BLA. Unfortunately, due to the presence of both NE and DA in the CA signal, as evidenced by idazoxan and raclopride increasing the measured efflux, it is not currently possible to disentangle the effects of alcohol on NE or DA in the stressed subjects. However, it is notable that when only NE was evoked in the BLA following LC stimulation, alcohol did not affect the measured signal. These findings may suggest that CA efflux decreased after stress because alcohol only influenced the DA portion. In fact, divergent effects of alcohol on NE and DA transmission under normal conditions have been observed in some brain areas. For example, it was found using FSCV that LC-induced NE in the mPFC is not affected by acute alcohol, while VTA-induced DA in the same brain region is decreased by alcohol (Shnitko et al., 2014; Deal et al., 2019). Moreover, a microdialysis study revealed that alcohol (2 g/kg) increased extracellular DA but not NE in the BLA of group housed rats (Karkhanis et al., 2015). As discussed previously (Jones et al., 2006), an increase in DA levels, as measured by microdialysis, would be consistent with a decrease in the amount of electrically-evoked DA detected with FSCV *in vivo*. This may be simply due to autoreceptor feedback induced by increased cell firing rates and accumulating DA concentrations in terminals. Further, the effect of acute alcohol on extracellular DA concentrations can be sensitized in the BLA following stress through adolescent social isolation (Karkhanis et al., 2015). Therefore, there are multiple studies which provide indirect evidence to support the involvment of DA in the effect of alcohol on evoked CA release in the BLA.

However, we cannot exclude the role of NE in this effect. Indeed, while extracellular NE concentrations were unchanged by alcohol under normal conditions, social isolation stress resulted in a significant increase in this measure (Karkhanis et al., 2015). Strikingly, alcohol-induced increases in both NE and DA levels measured by microdialysis exhibited a similar time course to the decrease in electrically-evoked CA release mesured by voltammetry. Taken together, the current and previous findings support the hypothesis that alcohol may be differently targeting DA and NE release in the BLA following stress exposure.

In conclusion, we have shown that stress, whether social or non-social, can lead to lasting alterations in CA activity that, in turn, affect how alcohol influences CA release in the BLA. Stress changes the LC-induced CA landscape of the BLA, introducing a locus of effect for alcohol that is not present in non-stressed subjects.

## Data Availability Statement

The raw data supporting the conclusions of this article will be made available by the authors, without undue reservation.

## Ethics Statement

The animal study was reviewed and approved by Wake Forest University School of Medicine Institutional Animal Care and Use Committee.

## Author Contributions

EB and JW designed the electrochemical and behavioral experiments. AD performed the electrochemical and behavioral tests and analyzed the data. JP conducted the histology. All authors contributed to the article and approved the submitted version.

## Conflict of Interest

The authors declare that the research was conducted in the absence of any commercial or financial relationships that could be construed as a potential conflict of interest.
